# Fisetin Attenuates Arsenic-Induced Hepatic Damage by Improving Biochemical, Inflammatory, Apoptotic, and Histological Profile: In Vivo and In Silico Approach

**DOI:** 10.1155/2022/1005255

**Published:** 2022-10-20

**Authors:** Muhammad Umar, Saima Muzammil, Muhammad Asif Zahoor, Shama Mustafa, Asma Ashraf, Sumreen Hayat, Muhammad Umar Ijaz

**Affiliations:** ^1^Department of Zoology, Wildlife and Fisheries, University of Agriculture, Faisalabad, Pakistan; ^2^Department of Microbiology, Government College University, Faisalabad, Pakistan; ^3^Department of Zoology, Government College University, Faisalabad, Pakistan

## Abstract

Arsenic (As) is a toxic metalloid and human carcinogen that may cause hepatotoxicity. Fisetin (3, 3′, 4′, 7-tetrahydroxyflavone) is a phytoflavonoid, which shows diverse therapeutic activities. This study aimed to examine the remedial potential of fisetin against As-instigated hepatotoxicity in adult male rats. To accomplish this aim, albino rats (*N* = 48) were evenly classified into 4 groups: control group, As (10 mg/kg) group, fisetin (2.5 mg/kg) + As (10 mg/kg) group, and fisetin (2.5 mg/kg) group. After one month of treatment, biochemical assay, total protein content (TPC), hepatic serum enzymes, inflammatory as well as pro- or anti-apoptotic markers, and histopathological profile of hepatic tissues were estimated. As administration disordered the biochemical profile by decreasing activities of antioxidant enzymes i.e., catalase (CAT), superoxide dismutase (SOD), glutathione reductase (GSR), and glutathione (GSH) content while escalating the levels of reactive oxygen species (ROS), and thiobarbituric acid reactive substances (TBARS). TPC was also considerably reduced after exposure to As. Furthermore, As markedly raised the levels of liver serum enzymes such as aspartate transaminase (AST), alkaline phosphatase (ALP), and alanine transaminase (ALT) as well as the levels of inflammatory markers, i.e., nuclear factor- *κ*B (NF-*κ*B), tumor necrosis- *α* (TNF-*α*), interleukin-1*β* (IL-1*β*), interleukin-6 (IL-6), and cyclo-oxygenase-2 (COX-2) activity. Besides, it lowered the level of antiapoptotic markers (Bcl-2) and upregulated the levels of proapoptotic markers (Bax, Caspase-3, and Caspase-9). Additionally, As exposure led to histopathological damage in hepatic tissues. However, fisetin administration remarkably alleviated all the depicted hepatic damages. For further verification, the screening of several dock complexes was performed by using the GOLD 5.3.0 version. Based on docking fitness and GOLD score, the ranking order of receptor proteins with fisetin compound is superoxide dismutase, interleukin, aspartate aminotransferase, alkaline phosphatase, TNF-alpha, alanine transaminase, cyclo-oxygenase 2, antiapoptotic, and glutathione reductase. Out of these three receptor proteins superoxide dismutase, interleukin, and aspartate aminotransferase showed the best interaction with the fisetin compound. In vivo and in silico outcomes of the current study demonstrated that fisetin could potentially ameliorate As-instigated hepatotoxicity.

## 1. Introduction

Arsenic (As) is a noxious metalloid, which is ranked 1^st^ by the United States (US) Agency for Disease Registry and Toxic Substances as well as US Environmental Protection Agency [1] that affected nearly 200 million people globally [2]. The most reported types of As-instigated damages in humans include skin diseases (viz. hyperkeratosis, hyper-pigmentation), skin or epithelial tissues cancers; respiratory tract, gastrointestinal tract, liver, kidney, central nervous system, cardiovascular, and reproductive complexities, thereby enhancing the rate of morbidity and mortality [3]. Humans get exposed to arsenic via inhalation, skin contact with As-contaminated products, and polluted drinking water (H_2_O) [4]. Moreover, As toxicity depends on the chemical nature of arsenicals (arsenic-comprising compounds), which exist in both organic and inorganic forms with differently charged cations (e.g., As3þ and As5þ) [5]. Overall, the inorganic form of As is more toxic than the organic form of this metal [6].

After absorption from the lungs, As is delivered by the gut into the bloodstream where it (99% of arsenic) binds with red blood cells in circulating fluid, which eventually transports it to other parts of the body [7] and accumulates in different organs, i.e., lungs, liver, kidney, and heart [8]. The liver is a vital organ that tends to retain higher concentrations of As [9]. One of the most generally putative mechanisms to describe As-instigated toxicities is oxidative stress (OS) [10]. OS can cause mitochondrial dysfunction via fibrosis (TGF-*β*/Smad pathway), inflammation (NF-ĸB, TNF-*α*, IL-1, and IL-6), apoptosis (AKT-PKB, PI3/AKT, AKT/ERK, MAPK, PKC*δ*-JNK, and p53 pathways), and necrosis [11]. Besides, As intoxication was shown to weaken the antioxidant defense and damage several macromolecules (deoxyribonucleic acid (DNA), proteins, and lipids), which led to the foundation of the membrane, cell, and tissue dysfunction [12]. Moreover, As exposure may lead to inflammation which also results in liver damage. Thus, after scrutinizing the numerous sources of As exposure and their damaging impacts on human health, especially on the liver, a study on therapies against As-induced toxicities is needed.

The advantage of using in silico methods for drug design is that it takes less time and money to find novel targets. Several biological issues have been resolved using in silico techniques that can characterize interacting molecules and forecast three-dimensional (3D) structures. To ascertain how various target proteins interact with the discovered chemical, in silico investigation was carried out. In this instance, fisetin (3,3′,4′,7-tetrahydroxyflavone) is a phytoflavonoid, which profoundly exists in multiple dietary sources such as apple, persimmon, grape, strawberry, cucumber, onion, and its quantities range from 2 to 160 mg/g with an average everyday consumption estimation of 0.4 mg [13]. It shows a broad range of therapeutic activities that include antioxidant [14], anticarcinogenic [13], anti-inflammatory [15], neuroprotective [15], and cardioprotective effects [14]. Up till now, the ameliorative potential of fisetin against arsenic-provoked hepatotoxicity is not available. So, the present investigation proposed to explore the remedial potency of fisetin against As-instigated hepatotoxicity in rats.

## 2. Materials and Methods

### 2.1. Chemicals

As and fisetin were purchased from Germany (Sigma-Aldrich).

### 2.2. Animals

Sexually mature male albino rats (*n* = 48) weighing 150 ± 30 g were kept in 12 rats per cage (made of steel) in the animal breeding as well as rearing house of the University of Agriculture, Faisalabad. All the rats were provided with tap water ad libitum as well as standard chow and photoperiod of 12 h light/dark cycle at temperature ranges between 23 and 26°C. Rats were kept in subordination with the European Union protocol (CEE Council 86/609) of animal care and experimentation.

## 3. Experimental Protocol

Albino rats (*N* = 48) were allocated into 4 groups (*N* = 12) and administered orally the following: control group (Treated with normal saline), As group (10 mg/kg. b. wt. Of As), cotreated group (10 mg/kg b.wt. Of As and 2.5 mg/kg. b.wt. Of fisetin), and only fisetin administered group (2.5 mg/kg.b.wt. Of fisetin). The entire experimental trial was conducted for thirty days. After one month of treatment, hepatic tissues were excised, weighed, and kept till additional analysis.

### 3.1. Biochemical Assay and TPC

In the hepatic tissues, the activity of CAT was ascertained according to the methodology described by Chance and Maehly [16]. SOD activity was measured by following the process of Kakkar et al. [17]. GSR activity was determined according to the protocol of Carlberg and Mannervik [18]. GSH content was measured via the technique designed by Jollow et al. [19]. Hayashi et al. [20] protocol was used to estimate the level of ROS. The level of TBARS was assessed by following the technique of Iqbal et al. [21]. The TPC of hepatic tissues was quantified according to the Lowry method as modified by Peterson [22].

### 3.2. Liver Serum Enzymes

The levels of ALT, AST, and ALP were determined in accordance with the commercial kits purchased from Wiesbaden, Germany.

### 3.3. Inflammation

The levels of TNF-*α*, NF-*κ*B, IL-6, IL-1*β*, and COX-2 activity were estimated with an ELISA kit as per the company's guidance, BioTek, Winooski, VT, United States of America (USA).

### 3.4. Apoptosis

The levels of Bcl-2, Bax, Caspase-3, and Caspase-9 were estimated with the help of ELISA kits bought from Cusabio Technology Llc, Houston, TX, USA.

### 3.5. Histopathology

For histopathological analysis, initially, hepatic tissues were cleaned in 0.9% chilled saline and placed in 10% formalin solution, subsequently desiccated in mounting concentrations of alcohol, and embedded in paraffin wax. After that, paraffin-encased 5-µm slices were pruned via microtome, and staining was done with the help of hematoxylin-eosin (H&E) stain and observed below the Leica LB microscope at 400X [23].

### 3.6. Statistical Analysis

The results mean ± standard error (SE) was presented in the tables after applying ANOVA accompanied by Tukey's test to interpret the entire data with the help of Minitab software. Results were declared meaningful at *p* < 0.05.

### 3.7. In Silico Analysis

#### 3.7.1. Ligand Preparation

The two-dimensional (2D) configuration of fisetin phytocompound retrieved from PubChem (https://pubchem.ncbi.nlm.nih.gov) and treated in the ChemDraw ultra 12.0 and Chem 3D Pro for ionization, minimization, and optimization of ligands. Force field via the module for minimization and optimization of ligands having the lowest energy conformer of the ligand.

#### 3.7.2. Receptor Preparation

In order to assess the molecular docking, optimum resolution X-ray structures of proteins were obtained from the Protein Databank (RCSB PDB) (https://www.rcsb.org) and underwent the Protein preparation wizard of Maestro (Gold v 5.3.0). This module processed the protein by the addition of hydrogen atoms to the protein structure, removing solvent molecules (H2O), creating disulfide bonds, assigning bond orders, filling missing side chains as well as loops, and generating a protonation state at the cellular level pH (7.4 ± 0.5) using the Epik tool of protein structures for ligands. Following the processing of protein structures, the PDB ID of this 5YTO [24], 1ILR [25], 6WNG (10.2210/pdb6WNG/PDB), 1ANJ [26], 5YOY [27], IBDO [28], 51F9 [29], 6FSO [30], and 6TJL (10.2210/pdb6TJL/PDB) structures were optimized using GOLD at pH 7.0, and the OPLS3e force field was used to perform restrained minimization for energy minimization and protein structural geometry optimization.

#### 3.7.3. Molecular Docking

The docking studies were carried out with the use of molecular docking software parameters (https://www.ccdc.cam.ac.uk). Docking simulations were carried out by the Lamarckian genetic algorithm (LGA) and the Solis and Wets local search approach. The initial position, orientation, and torsion of the ligand molecules were determined at random. Every docking experiment was filtered from ten distinct runs that were set to stop followed by a maximum of 1.5 Å assessments.

Molecular docking experiments were used to investigate the potential binding/interaction between proteins and ligands.  Table S1 illustrates the binding affinity (kcal/mol) of the fisetin (5281614) phytocompound with different receptor proteins. Three-dimensional structures of receptor proteins, alkaline phosphate (PDB ID 1ANJ), alanine transaminase (PDB ID, IBDO), cyclooxygenase-2 (PDB ID 51F9), interleukin (PDB ID, 1ILR), TNF-a (PDB ID, 5YOY), superoxide dismutase (PDB ID, 5YTO), antiapoptotic (PDB ID, 6FSO), glutathione reductase (PDB ID, 6TJL), and aspartate aminotransferase (PDB ID, 6WNG) were acquired from the PDB database (Protein Data Bank). Docking calculations were carried out with GOLD version 5.3.0 and BIOVIA discovery studio (http://www.3dsbiovia.com) for modeling and visualization. The initial position, orientation, and torsion of the ligand molecules were determined at random. Every docking experiment was extracted from a total of ten distinct runs that were set to end after a maximum of 1.5 Å evaluations.

## 4. Results

### 4.1. Effect of Fisetin on Biochemical Assay and TPC

The activity of CAT, SOD, GSR, as well as GSH level and TPC, was substantially (*p* < 0.05) reduced after As intoxication, while the concentration of ROS and level of TBARS were raised as matched with the untreated group. Conversely, fisetin supplementation with As remarkably (*p* < 0.05) elevated the activity of CAT, SOD, GSR, and GSH content as well as TPC, while considerably (*p* < 0.05) lowered the levels of ROS and TBARS in the cotreated group as contrasted with the As-induced group. Nonetheless, nonsignificant variation was witnessed among rats of the fisetin-only administrated and the untreated rats (Table 1).

### 4.2. Effect of Fisetin on AST, ALP, and AST

Table 2 shows outcomes of the study exposed that hepatic serum levels of AST, ALP, as well as ALT, were substantially (*p* < 0.05) raised in the As-induced group as matched to the control group. Nevertheless, fisetin treatment substantially (*p* < 0.05) caused the decline of hepatic enzymes in the cotreated group as matched to the As-intoxicated group. Moreover, a nonsignificant variation was seen between the fisetin-only treated and the control groups.

### 4.3. Effect of Fisetin on Inflammatory Markers

Table 3 demonstrates the outcomes of the investigation that displayed As exposure substantially (*p* < 0.05) raised the levels of IL-1*β*, TNF-*α*, NF-*κ*B, IL-6, and COX-2 activity in the As-induced group as matched to the control group. Nonetheless, fisetin supplementation markedly (*p* < 0.05) diminished inflammatory indices in the cotreated (As + fisetin) group as matched to the As group. There was insignificant (*p* < 0.05) variation between the fisetin-only treated and the control groups.

### 4.4. Effect of Fisetin on Antiapoptotic and Proapoptotic Markers

To ascertain the probable antiapoptotic activity of fisetin, a property that presents its protecting impact against As-instigated hepatic tissues apoptosis, we estimated the alterations in the levels of the antiapoptotic marker Bcl-2 and proapoptotic markers, particularly, Bax, Caspase-3, and Caspase-9 (Table 4). Results of the study exposed that As-induction considerably (*p* < 0.05) decreased the antiapoptotic indices, whereas increased the proapoptotic inducers in the As-intoxicated rats as matched with the control rats. Nevertheless, fisetin cotreatment substantially (*p* < 0.05) restored the level of the above-stated antiapoptotic marker while reducing the levels of proapoptotic markers in the cotreated group as contrasted with the arsenic-induced group. However, a nonsignificant alteration was noticed among the mean values of the fisetin-only treated and the untreated rats.

### 4.5. Effect of Fisetin on Histopathology

**Figure 1:**
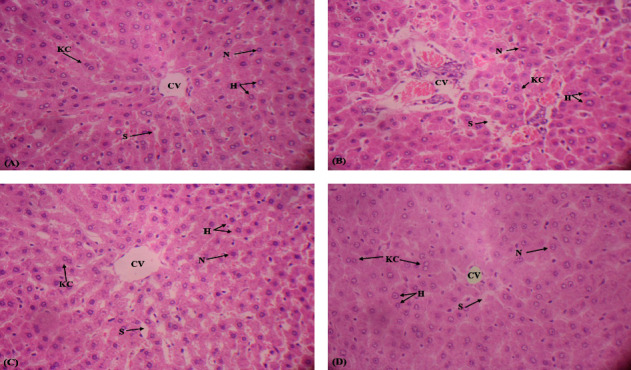
Protecting impact of fisetin on arsenic deteriorated histopathology (hematoxylin-eosin. 40X). (a) Control group; (b) arsenic group (50 mg/kg); (c) arsenic (50 mg/kg) + fisetin group (50 mg/kg); (d) fisetin group (50 mg/kg). Central venule (CV); Kupffer cells (KC); hepatocytes (H); sinusoids (S); nucleus (N).

Figure 1 shows the comparative changes in the histopathological profile. Outcomes of the investigation presented that As exposure caused necrosis, sinusoid dilation, and apoptosis of hepatocytes along with central venule disruption in the As-induced rats as matched to the control rats (Figures 1(b) and 1(a)). Nonetheless, fisetin supplementation substantially (*p* < 0.05) mitigated the predominance and intensity of histopathological impairments such as diminished dilation of sinusoids with no necrotic cell, central venule disruption, and retrieved the classic architecture of liver cells in the coadministrated (As + fisetin) group as matched to the As group (Figures 1(b) and 1(c)). However, in the fisetin-only treated rats, histological architecture was similar to the control group (Figures 1(d) and 1(a)).

## 5. Discussion

Arsenic as a poison is a worldwide health predicament. Chronic As intoxication has been profoundly linked with several disorders and health problems in humans [31]. The overgeneration of intracellular ROS after As exposure mediates multiple alterations in cell functioning by changing signaling molecular antigenic alteration or provokes direct oxidative impairment to molecules [32]. Thus, antioxidants with remarkable free radical scavenging properties can alleviate As-instigated toxicities [33]. Therefore, the current investigation was formulated to estimate the antioxidant potency of fisetin, which is a potential flavonoid with diverse pharmacological properties against As-intoxicated hepatotoxicity in rats.

Outcomes of the current research revealed that As intoxication considerably reduced activities of SOD, CAT, GSR, or GSH content, and TPC while escalating the levels of ROS and TBARS. As evident, the body's defense system is made up of antioxidants that may be enzymatic or nonenzymatic, which act swiftly and neutralize free radicals [34]. SOD, GPX, and CAT are enzymatic antioxidants, while GSH is a nonenzymatic antioxidant [35]. CAT and GPx transformed the hydrogen peroxide (H_2_O_2)_ into the water [36], whereas the conversion of superoxide anion (O_2_^−^) into H_2_O_2_ is carried out by SOD [37] whereas reduced GSH functions as an anion donator in these redox reactions [38]. GSH is retained by GSR, which renovates reduced GSH from oxidized GSSG for the perpetual functioning of GPx [39]. However, an unnecessary escalation of ROS resulting from deficient antioxidant defense or collapse of the cells' buffering system to retain the redox balance that leads to OS, which consequently commences numerous modifications in biomolecules and ultimately leads to disease conditions [40]. The oxidative damage to lipids is known as LPO [41]. LPO, in turn, may lead to damages that affect membrane integrity as well as fluidity and permeability [42]. However, fisetin provision remarkably alleviated the above-stated biochemical alteration via enhancing the activities of antioxidant defense or total protein content and lowering the levels of ROS and TBARS. This curative effect of fisetin may be due to the presence of one hydroxyl group on its A-ring that sets the lipid-H_2_O interface of the membrane and exhibits equivalent free radical scavenging activity similar to other flavonoids such as quercetin. Thus, it inhibited LPO by preventing the additional diffusion of reactive oxygen species into the lipid hydrophobic core [8].

In the current investigation, As exposure caused a remarkable increment in ALP, ALT, and AST levels indicating damage to hepatic tissues. As documented earlier, these enzymes exist in hepatocytes, but their levels are ordinarily low. However, when liver cells are damaged, their membranes become more penetrable as a result their enzymes are liberated into the blood [43]. Our outcomes are in harmony with the results of Un et al. [44], who conveyed similar results, followed by As treatment. However, in the current research, fisetin oral gavage remarkably reduced the levels of hepatic serum enzymes, which may be due to its antioxidant potential.

Inflammation is the reflective response of the body's defense system, which is provoked by internal, i.e., stressed, impaired, or defective functioning of tissues, as well as external sources, i.e., reactive chemicals, allergens, microbes, and ROS [45]. This inflammatory process leads to elevated cell membrane permeability and vasodilatation that causes the nuclear translocation of different leukocytes and inflammatory markers [46]. NF-*κ*B is among the fundamental inflammatory mediators which get triggered instantly in response to the internal or external cellular stimulant, which ultimately increases the levels of TNF-*α* [46], IL-1*β* [47], IL-6 [46] and activity of COX-2 [47]. Outcomes of the present investigation showed that As-induction substantially boosted the levels of IL-1*β*, TNF-*α*, NF-*κ*B, IL-6, and COX-2 activity. However, fisetin coadministration with As remarkably lowered the elevated levels of inflammatory markers, which showed its anti-inflammatory property.

Apoptosis, a cell death mechanism, which helps to eradicate undesired cells, is accomplished by intrinsic (mitochondrial) and extrinsic (death receptor) pathways [48]. In the current investigation, we assessed apoptosis by estimating the level of Bax, Caspase-3, Caspase-9, and Bcl-2. Outcomes showed that As exposure lowered the level of Bcl-2 while boosting the levels of Bax, Caspase-3, and Caspase-9. Bax and Bcl-2 are proteins that are related to the Bcl-2 family. Bcl-2 promotes cellular longevity by stabilizing the opening of the MPT (mitochondrial-permeability-transition) pore complex and defends against Cytochrome *c* liberation, whereas Bax activates MPT pore and regulates the discharge of Cytochrome *c* into the cytosol [49], which activate Caspase-9 that cleaves Caspase-3 [50], that eventually leads to apoptosis [51]. As evident, Caspases are cysteine proteases that cut 100 distinct target proteins and provoke apoptosis [52]. Thus, the anti- or pro-apoptotic Bcl-2/Bax ratio regulates apoptosis [53]. Nevertheless, fisetin mitigated these hepatocytes' apoptosis via down-and-upregulating the levels of pro- or anti-apoptotic markers, respectively, in the rat liver. Our outcomes verify the antiapoptotic potential of fisetin.

The outcomes of the present research demonstrated that As administration induced intense histopathological impairments in the hepatic tissues. The reason behind these toxic histological alterations is LP, which eventually leads to inflammation and apoptosis in hepatocytes. As-induced hepatic injuries include central venule disruption, apoptosis of hepatocytes, necrosis, and sinusoid dilation. Our results are compatible with Al-Forkan et al. [54], who studied the retention mechanism of As in organs and its effect on liver enzymes, hematology, and histology. However, fisetin treatment remarkably ameliorated the histopathological damages caused by As. Fisetin restored the impairments of its histopathological profile may be due to its free radical quenching, anti-inflammatory, and antiapoptotic, attributes.

### 5.1. Docking Analysis

ChemDraw Ultra 12.0 and Chem 3D Pro were utilized in GOLD docking for the energy minimization of ligands in accordance with the method adopted by Andleeb et al. (2020). In order to comprehend the efficacy of these receptors, the molecular docking analysis looked at the molecular interactions of the fisetin molecule with different receptor proteins. The protein data repository was considered to obtain the coordinate crystal structure of the receptor proteins (PDB). The GOLD suite version 5.3.0 with a high resolution of 2.70 was then used to load each of these structures one at a time for docking. Using the GOLD 5.3.0 edition, screenings of various dock complexes were done based on docking fitness and GOLD score. The most effective chemical that interacts with the receptor was discovered thanks to the GOLD program. Based on docking score and fitness, the results were evaluated for binding compatibility. The ligand molecule with the highest binding affinity to the receptor molecule was selected as the best medication.

**Figure 2:**
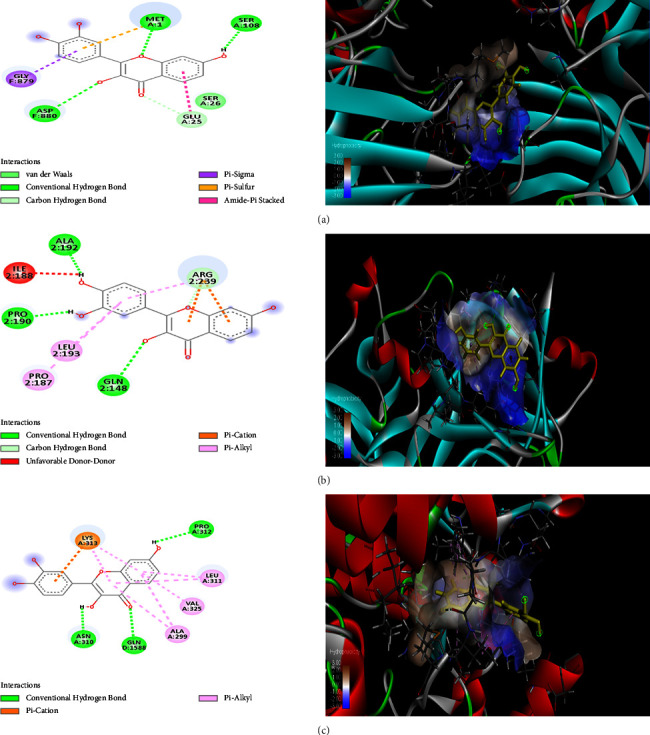
In silico molecular docking analysis of 2D and 3D interactions among fisetin and screened receptor proteins (a) 5YTO, (b) 1ILR, and (c) 6WNG.

The three receptor proteins 5YTO, 1ILR, and 6WNG demonstrated the best interaction with the fisetin compound, with GOLD fitness values of 77.99, 68.50, and 60.35 and GOLD docking scores of -9.29, -8.96, and -8.90, respectively. These interactions included the formation of a hydrogen bond (MET A:1, ASP A: 880, SER A: 108, GLU A:25, GLN A:148, and PRO A:190). These three receptor proteins displayed an incredibly strong association with the fisetin chemical and can be thought of as possible receptor molecules that may be useful as an indicator of inflammation, antiapoptotic activity, and antioxidant activity. With GOLD fitness values of 52.44, 53.42, 38.15, and 62.30, GOLD docking scores of -8.62, -8.42, -7.78, and -7.80, and interactions of the hydrogen bond with other molecules, 1ANJ, 5YOY, IBDO, and 51F9 demonstrated moderate binding affinity (SER A: 99, ALA A: 321, ASP A: 98, ASP A: 135, ASP A: 728, PRO A: 134, GLN A: 148, PRO A: 190, ALA A: 192, ARG A: 239, PRO A: 187, THR A: 181, ASN A: 351, HIS A: 357, THR A: 175, and PHE A: 179). The interaction between 6FSO and 6TJL is the least favorable, falling between 53.04 and 50.93, with docking scores of -7.19 and -7.32. The receptor proteins with fisetin compound are ranked in the following order: 5YTO > 1ILR > 6WNG > 1ANJ > 5YOY > IBDO > 51F9 > 6FSO > 6TJL. The best poses created by the discovery studio are depicted in Figure 2 in a 2D depiction of the interactions between proteins and ligands.

## 6. Conclusion

Adult male albino rats received the arsenic injection, which resulted in elevated serum enzyme levels, inflammatory and apoptotic indicators, and a worsened histopathological profile. Additionally, an unbalanced state that resulted in hepatic dysfunction was presented by the activity of enzymatic antioxidants, TPC or the levels of ROS, and TBARS. Nevertheless, due to its underlying antioxidant, antiapoptotic, as well as anti-inflammatory potentials, fisetin therapy significantly reduced arsenic-induced deficits in all of the aforementioned measures. [55]

## Figures and Tables

**Table 1:** Presents the outcomes of biochemical analysis along with total protein content.

Groups	Control	Arsenic	Arsenic + Fisetin	Fisetin
CAT (U/mg protein)	7.62 ± 0.09^a^	3.58 ± 0.26^b^	6.77 ± 0.06^a^	6.99 ± 0.07^a^
SOD (U/mg tissue)	6.34 ± 0.12^a^	3.29 ± 0.09^b^	5.65 ± 0.06^a^	6.19 ± 0.11^a^
GSR (nm NADPH oxidized/min/mg tissue	3.03 ± 0.06^a^	1.62 ± 0.16^b^	2.66 ± 0.06^a^	2.96 ± 0.05^a^
GSH (nM/min/mg protein)	15.87 ± 0.21^a^	8.99 ± 0.121^b^	14.98 ± 0.07^a^	15.17 ± 0.09^a^
ROS (U/mg tissue)				
TBARS (nM/min/mg tissue)	14.47 ± 0.2^a^	22.750 ± 0.27^b^	15.91 ± 0.07^a^	15.16 ± 0.19^a^
Total protein (*µ*g/mg tissues)	4.04 ± 0.08^a^	1.88 ± 0.06^b^	3.85 ± 0.04^a^	4.02 ± 0.07^a^

Superscripts indicate considerable difference at probability value *p* < 0.05.

**Table 2:** Displays the levels of liver serum enzymes.

Groups	Control	Arsenic	Arsenic + Fisetin	Fisetin
ALP (U/I)	67.00 ± 2.5^a^	176.33 ± 5.21^b^	101.7 ± 1.77^a^	86.67 ± 2.97^a^
ALT (U/I)	42.33 ± 4.4^a^	243.00 ± 7.24^b^	76.33 ± 6.94^a^	54.00 ± 3.79^a^
AST (U/I)	65.00 ± 2.9^a^	279.67 ± 11.5^b^	106.3 ± 2.41^a^	78.33 ± 5.05^a^

Superscripts indicate considerable difference at probability value *p* < 0.05.

**Table 3:** Depicts levels of inflammatory markers.

Groups	Control	Arsenic	Arsenic + Fisetin	Fisetin
NF-*κ*B (ng/g tissue)	12.53 ± 0.64^a^	64.38 ± 0.94^b^	17.97 ± 1.00^a^	12.16 ± 0.67^a^
TNF-*α* (ng/g tissue)	6.31 ± 0.53^a^	17.76 ± 1.02^b^	8.73 ± 0.50^a^	6.26 ± 0.56^a^
IL-1*β* (ng/g tissue)	23.89 ± 1.13^a^	87.38 ± 1.28^b^	29.57 ± 1.09^a^	23.84 ± 1.01^a^
IL-6 (ng/g tissue)	4.62 ± 0.45^a^	22.64 ± 2.00^b^	7.11 ± 0.98^a^	4.59 ± 0.37^a^
COX-2 (ng/g tissue)	23.39 ± 0.80^a^	65.75 ± 2.19^b^	29.10 ± 1.29^a^	23.36 ± 0.70^a^

Superscripts indicate considerable difference at probability value *p* < 0.05.

**Table 4:** Shows the levels of proapoptotic and antiapoptotic markers.

Groups	Control	Arsenic	Arsenic + Fisetin	Fisetin
Bcl-2	14.49 ± 0.65^a^	6.10 ± 0.98^b^	12.34 ± 0.28^a^	14.57 ± 0.69^a^
Bax	2.58 ± 0.25^a^	7.52 ± 0.34^b^	2.92 ± 0.21^a^	2.55 ± 0.12^a^
Caspase-3	1.73 ± 0.09^a^	10.58 ± 0.57^b^	2.74 ± 0.31^a^	1.71 ± 0.12^a^
Caspase-9	4.70 ± 0.18^a^	15.24 ± 0.79^b^	5.81 ± 0.25^a^	4.69 ± 0.21^a^

Superscripts indicate considerable difference at probability value *p* < 0.05.

## Data Availability

The data supporting the current study are available from the corresponding author upon request.

## Supplementary Materials

Supplementary Materials Table S1 illustrates the binding affinity (kcal/mol) of the fisetin (5281614) phytocompound with different receptor proteins.

## References

[1] Agency for Toxic Substances and Disease Registry (2017). *Priority Substance List*.

[2] Coryell M., McAlpine M., Pinkham N. V., McDermott T. R., Walk S. T. (2018). The gut microbiome is required for full protection against acute arsenic toxicity in mouse models. *Nature Communications*.

[3] Mazumder D. G. (2008). Chronic arsenic toxicity and human health. *Indian Journal of Medical Research*.

[4] Shi H., Hudson L. G., Ding W. (2004). Arsenite causes DNA damage in keratinocytes via generation of hydroxyl radicals. *Chemical Research in Toxicology*.

[5] Rubin D. C., Alava S. S., Zekker P., Du Laing I., Van de Wiele T. (2014). Arsenic thiolation and the role of sulfate-reducing bacteria from the human intestinal tract. *Environmental Health Perspectives*.

[6] Jomova K., Jenisova Z., Feszterova M. (2011). Arsenic: toxicity, oxidative stress and human disease. *Journal of Applied Toxicology*.

[7] Roshni P. R., Aiswarya P., Remya R., Meenu V. (2015). Environmental and occupational risk factors associated with lung cancer. *World Journal of Pharmacy and Pharmaceutical Sciences*.

[8] Kaur T., Singh A., Goel R. (2014). Mechanisms pertaining to arsenic toxicity. *Toxicology International*.

[9] Renu K., Saravanan A., Elangovan A. (2020). An appraisal on molecular and biochemical signalling cascades during arsenic-induced hepatotoxicity. *Life Sciences*.

[10] Li C., Li P., Tan Y. M., Lam S. H., Chan E. C. Y., Gong Z. (2016). Metabolomic characterizations of liver injury caused by acute arsenic toxicity in zebrafish. *PLoS One*.

[11] Renu K., Saravanan A., Elangovan A. (2020). An appraisal on molecular and biochemical signalling cascades during arsenic-induced hepatotoxicity. *Life Sciences*.

[12] Muthumani M., Prabu S. M. (2012). Silibinin potentially protects arsenic-induced oxidative hepatic dysfunction in rats. *Toxicology Mechanisms and Methods*.

[13] Imran M., Saeed F., Gilani S. A. (2021). Fisetin: an anticancer perspective. *Food Sciences and Nutrition*.

[14] Sharma U. R., Sowparnika E., Vada S., Taj N., Mudagal M. P. (2020). Preventive effect of Fisetin on Cardiac markers, Lipid peroxides and Antioxidants in normal and Ischemia-Reperfusion induced Myocardial infarction in rats. *International Journal of Biological & Pharmaceutical Research*.

[15] Naeimi A. F., Alizadeh M. (2017). Antioxidant properties of the flavonoid fisetin: an updated review of in vivo and in vitro studies. *Trends in Food Science & Technology*.

[16] Chance B., Maehly A. (1955). 136] assay of catalases and peroxidases. *Methods in Enzymology*.

[17] Kakkar P., Das B., Viswanathan P. N. (1984). A modified spectrophotometric assay of superoxide dismutase. *Indian Journal of Biochemistry and Biophysics*.

[18] Carlberg I., Mannervik B. (1975). Purification and characterization of the flavoenzyme glutathione reductase from rat liver. *Journal of Biological Chemistry*.

[19] Jollow D. J., Mitchell J. R., Zampaglione N. A., Gillette J. R. (1974). Bromobenzene-induced liver necrosis. Protective role of glutathione and evidence for 3, 4-bromobenzene oxide as the hepatotoxic metabolite. *Pharmacology*.

[20] Hayashi I., Morishita Y., Imai K., Nakamura M., Nakachi K., Hayashi T. (2007). High-throughput spectrophotometric assay of reactive oxygen species in serum. *Mutation Research/Genetic Toxicology and Environmental Mutagenesis*.

[21] Iqbal M., Sharma S. D., Rezazadeh H., Hasan N., Abdulla M., Athar M. (1996). Glutathione metabolizing enzymes and oxidative stress in ferric nitrilotriacetate mediated hepatic injury. *Redox Report*.

[22] Peterson G. L. (1977). A simplification of the protein assay method of Lowry et al. which is more generally applicable. *Analytical Biochemistry*.

[23] Fukuzawa Y., Watanabe Y., Inaguma D., Hotta N. (1996). Evaluation of glomerular lesion and abnormal urinary findings in OLETF rats resulting from a long-term diabetic state. *The Journal of Laboratory and Clinical Medicine*.

[24] Manjula R., Wright G. S. A., Strange R. W., Padmanabhan B. (2018). Assessment of ligand binding at a site relevant to SOD 1 oxidation and aggregation. *FEBS Letters*.

[25] Schreuder H. A., Rondeau J. M., Tardif C. (1995). Refined crystal structure of the interleukin‐1 receptor antagonist: presence of a disulfide link and a cis‐proline. *European Journal of Biochemistry*.

[26] Murphy J. E., Tibbitts T. T., Kantrowitz E. R. (1995). Mutations at positions 153 and 328 inEscherichia coliAlkaline phosphatase provide insight towards the structure and function of mammalian and yeast alkaline phosphatases. *Journal of Molecular Biology*.

[27] Ono M., Horita S., Sato Y., Nomura Y., Iwata S., Nomura N. (2018). Structural basis for tumor necrosis factor blockade with the therapeutic antibody golimumab. *Protein Science*.

[28] Stamper C. G. F., Morollo A. A., Ringe D. (1998). Reaction of alanine racemase with 1-aminoethylphosphonic acid forms a stable external aldimine. *Biochemistry*.

[29] Lucido M. J., Orlando B. J., Vecchio A. J., Malkowski M. G. (2016). Crystal structure of aspirin-acetylated human cyclooxygenase-2: insight into the formation of products with reversed stereochemistry. *Biochemistry*.

[30] Tron A. E., Belmonte M. A., Adam A. (2018). Discovery of Mcl-1-specific inhibitor AZD5991 and preclinical activity in multiple myeloma and acute myeloid leukemia. *Nature Communications*.

[31] Uygur R., Aktas C., Caglar V., Uygur E., Erdogan H., Ozen O. A. (2016). Protective effects of melatonin against arsenic-induced apoptosis and oxidative stress in rat testes. *Toxicology and Industrial Health*.

[32] Abdollahzade N., Majidinia M., Babri S. (2021). Melatonin: a pleiotropic hormone as a novel potent therapeutic candidate in arsenic toxicity. *Molecular Biology Reports*.

[33] Hu Y., Li J., Lou B. (2020). The role of reactive oxygen species in arsenic toxicity. *Biomolecules*.

[34] Majumder D., Nath P., Debnath R., Maiti D. (2021). Understanding the complicated relationship between antioxidants and carcinogenesis. *Journal of Biochemical and Molecular Toxicology*.

[35] Marrocco I., Altieri F., Peluso I. (2017). Measurement and clinical significance of biomarkers of oxidative stress in humans. *Oxidative Medicine and Cellular Longevity*.

[36] Aslani B. A., Ghobadi S. (2016). Studies on oxidants and antioxidants with a brief glance at their relevance to the immune system. *Life Sciences*.

[37] Ighodaro O. M., Akinloye O. A. (2018). First line defence antioxidants-superoxide dismutase (SOD), catalase (CAT) and glutathione peroxidase (GPX): their fundamental role in the entire antioxidant defence grid. *Alexandria Journal of Medicine*.

[38] Birben E., Sahiner U. M., Sackesen C., Erzurum S., Kalayci O. (2012). Oxidative stress and antioxidant defense. *World Allergy Organization Journal*.

[39] Ali S. S., Ahsan H., Zia M. K., Siddiqui T., Khan F. H. (2020). Understanding oxidants and antioxidants: classical team with new players. *Journal of Food Biochemistry*.

[40] Mukherjee K., Chio T. I., Sackett D. L., Bane S. L. (2015). Detection of oxidative stress-induced carbonylation in live mammalian cells. *Free Radical Biology and Medicine*.

[41] Gahalain N., Chaudhary J., Kumar A., Sharma S., Jain A. (2011). Lipid peroxidation: an overview. *International Journal of Pharmaceutical Sciences and Research*.

[42] Rawat N., Singla‐Pareek S. L., Pareek A. (2021). Membrane dynamics during individual and combined abiotic stresses in plants and tools to study the same. *Physiologia Plantarum*.

[43] Knudsen A. R., Andersen K. J., Hamilton‐Dutoit S., Nyengaard J. R., Mortensen F. V. (2016). Correlation between liver cell necrosis and circulating alanine aminotransferase after ischaemia/reperfusion injuries in the rat liver. *International Journal of Experimental Pathology*.

[44] Un H., Ugan R. A., Kose D. (2020). A novel effect of Aprepitant: protection for cisplatin-induced nephrotoxicity and hepatotoxicity. *European Journal of Pharmacology*.

[45] Jialal I., Pahwa R. (2019). Fetuin-A is also an adipokine. *Lipids in Health and Disease*.

[46] Khan I., Ullah N., Zha L. (2019). Alteration of gut microbiota in inflammatory bowel disease (IBD): cause or consequence? IBD treatment targeting the gut microbiome. *Pathogens*.

[47] Taniguchi K., Karin M. (2018). NF-*κ*B, inflammation, immunity and cancer: coming of age. *Nature Reviews Immunology*.

[48] Venkatadri R., Muni T., Iyer A. K. V., Yakisich J. S., Azad N. (2016). Role of apoptosis-related miRNAs in resveratrol-induced breast cancer cell death. *Cell Death and Disease*.

[49] Shalini S., Dorstyn L., Dawar S., Kumar S. (2015). Old, new and emerging functions of caspases. *Cell Death and Differentiation*.

[50] Kuida K. (2000). Caspase-9. *The International Journal of Biochemistry and Cell Biology*.

[51] Kaur S., Singh G., Sadwal S., Aniqa A. (2020). Alleviating impact of hydroethanolic Murraya koenigii leaves extract on bisphenol A instigated testicular lethality and apoptosis in mice. *Andrologia*.

[52] Coşkun G., Özgür H. (2011). Apoptoz ve nekrozun moleküler mekanizması. *Arşiv Kaynak Tarama Dergisi*.

[53] Zhao Q., Liu Y., Zhong J. (2019). Pristimerin induces apoptosis and autophagy via activation of ROS/ASK1/JNK pathway in human breast cancer in vitro and in vivo. *Cell death discovery*.

[54] Al-Forkan M., Islam S., Akter R. (2016). A sub-chronic exposure study of arsenic on hematological parameters, liver enzyme activities, histological studies and accumulation pattern of arsenic in organs of Wistar albino rats. *Journal of Cytology and Histology S*.

[55] Sinha R., Srivastava S., Joshi A., Joshi U. J., Govil G. (2018). In-vitroanti-proliferative and anti-oxidant activity of galangin, fisetin and quercetin: role of localization and intermolecular interaction in model membrane. *European Journal of Medicinal Chemistry*.

