# Supplementary material for: Fisetin Attenuates Arsenic-Induced Hepatic Damage by Improving Biochemical, Inflammatory, Apoptotic, and Histological Profile: In Vivo and In Silico Approach
Source: Evid Based Complement Alternat Med. 2022 Oct 20;2022:1005255. doi: 10.1155/2022/1005255 (PMC9613387; doi:10.1155/2022/1005255)
Supplement: Supplementary Materials — Table S1 illustrates the binding affinity (kcal/mol) of the fisetin (5281614) phytocompound with different receptor proteins. [file 1005255.f1.docx]

**Table S1**illustrates the binding affinity (kcal/mol) of the fisetin ([5281614](https://pubchem.ncbi.nlm.nih.gov/compound/5281614)) phytocompound with different receptor proteins**.**

| **Sr. NO** | **Receptor** |  | **GOLD Score**  **(kcal/mol)** | **Gold binding fitness**  **(Kcal/mol)** | **RMSD** | **Amino acid residue** | **Distance** | **Bond angle** |
| --- | --- | --- | --- | --- | --- | --- | --- | --- |
| **1.** | **1ANJ** |  | -8.62 | 52.44 | 1.81 | SER99 | 2.21 | Hydrogen Bond |
|  |  |  |  |  |  | ALA321 | 2.60 | Hydrogen Bond |
|  |  |  |  |  |  | ASP98 | 2.11 | Hydrogen Bond |
|  |  |  |  |  |  | ARG163 | 3.15 | Electrostatic |
|  |  |  |  |  |  | HIS328 | 5.56 | Hydrophobic |
| **2.** | **IBDO** |  | -7.78 | 38.15 | 0.30 | GLN148 | 2.38 | Hydrogen Bond |
|  |  |  |  |  |  | PRO190 | 1.54 | Hydrogen Bond |
|  |  |  |  |  |  | ALA192 | 1.39 | Hydrogen Bond |
|  |  |  |  |  |  | ARG239 | 2.47 | Hydrogen Bond |
|  |  |  |  |  |  | PRO187 | 5.45 | Hydrophobic |
|  |  |  |  |  |  | LEU193 | 5.28 | Hydrophobic |
|  |  |  |  |  |  | ARG239 | 5.45 | Hydrophobic |
| **3.** | **51F9** |  | -7.80 | 62.30 | 0.16 | THR181 | 2.91 | Hydrogen Bond |
|  |  |  |  |  |  | ASN351 | 2.25 | Hydrogen Bond |
|  |  |  |  |  |  | HIS357 | 2.40 | Hydrogen Bond |
|  |  |  |  |  |  | THR175 | 2.32 | Hydrogen Bond |
|  |  |  |  |  |  | PHE179 | 2.06 | Hydrogen Bond |
|  |  |  |  |  |  | HIS176 | 2.83 | Other |
|  |  |  |  |  |  | ALA171 | 3.96 | Hydrophobic |
| **4.** | **1ILR** |  | -8.96 | 68.50 | 0.74 | GLN148 | 2.38 | Hydrogen Bond |
|  |  |  |  |  |  | PRO190 | 1.54 | Hydrogen Bond |
|  |  |  |  |  |  | ALA192 | 1.39 | Hydrogen Bond |
|  |  |  |  |  |  | ARG239 | 2.47 | Hydrogen Bond |
|  |  |  |  |  |  | PRO187 | 5.45 | Hydrophobic |
|  |  |  |  |  |  | LEU193 | 5.28 | Hydrophobic |
|  |  |  |  |  |  | ARG239 | 5.45 | Hydrophobic |
| **5.** | **5YOY** |  | -8.42 | 53.42 | 1.03 | ASP135 | 2.23 | Hydrogen Bond |
|  |  |  |  |  |  | ASP728 | 1.49 | Hydrogen Bond |
|  |  |  |  |  |  | PRO134 | 1.97 | Hydrogen Bond |
|  |  |  |  |  |  | ARG133 | 3.63 | Electrostatic |
|  |  |  |  |  |  | ASP135 | 4.11 | Electrostatic |
|  |  |  |  |  |  | TYR710 | 3.24 | Hydrogen Bond |
|  |  |  |  |  |  | SER709 | 2.63 | Other |
|  |  |  |  |  |  | TYR708 | 4.05 | Hydrophobic |
| **6.** | **5YTO** |  | -9.29 | 77.99 | 0.14 | MET1 | 1.90 | Hydrogen Bond |
|  |  |  |  |  |  | ASP880 | 2.95 | Hydrogen Bond |
|  |  |  |  |  |  | SER108 | 1.69 | Hydrogen Bond |
|  |  |  |  |  |  | GLU25 | 2.52 | Hydrogen Bond |
|  |  |  |  |  |  | GLY879 | 2.89 | Hydrophobic |
|  |  |  |  |  |  | SER26 | 4.08 | Hydrophobic |
| **7.** | **6FSO** |  | -7.19 | 53.04 | 0.30 | ARG98 | 1.87 | Hydrogen Bond |
|  |  |  |  |  |  | MET85 | 2.90 | Hydrogen Bond |
|  |  |  |  |  |  | PHE89 | 2.37 | Hydrogen Bond |
|  |  |  |  |  |  | LEU102 | 2.60 | Hydrogen Bond |
|  |  |  |  |  |  | PHE105 | 4.95 | Hydrophobic |
| **8.** | **6TJL** |  | -7.32 | 50.93 | 0.25 | GLN40 | 2.71 | Hydrogen Bond |
|  |  |  |  |  |  | SER46 | 1.56 | Hydrogen Bond |
|  |  |  |  |  |  | ARG149 | 1.68 | Hydrogen Bond |
|  |  |  |  |  |  | LYS182 | 2.49 | Hydrogen Bond |
|  |  |  |  |  |  | MET99 | 2.71 | Hydrogen Bond |
|  |  |  |  |  |  | ALA44 | 2.70 | Hydrogen Bond |
|  |  |  |  |  |  | SER46 | 2.52 | Hydrogen Bond |
|  |  |  |  |  |  | HIS159 | 2.98 | Hydrophobic |
| **9.** | **6WNG** |  | -8.90 | 60.35 | 0.34 | GLN1588 | 2.97 | Hydrogen Bond |
|  |  |  |  |  |  | ASN310 | 1.83 | Hydrogen Bond |
|  |  |  |  |  |  | PRO312 | 2.25 | Hydrogen Bond |
|  |  |  |  |  |  | ALA299 | 5.41 | Hydrophobic |
|  |  |  |  |  |  | LEU311 | 5.41 | Hydrophobic |
|  |  |  |  |  |  | VAL325 | 4.85 | Hydrophobic |
|  |  |  |  |  |  | LYS313 | 5.24 | Hydrophobic |
